# The Atlanta Quarantine Convention

**Published:** 1898-05

**Authors:** 


### EDITORIAL.

The office of THE JOURNAL is 311 and 312 Fitten Building.

Address all communications, and make all remittances payable to THE ATLANTA MEDICAL AND SURGICAL JOURNAL, Atlanta, Ga.

Reprints of articles will be furnished, when desired, at a small cost. Requests for the same should always be made *on the manuscript*.

### THE ATLANTA QUARANTINE CONVENTION.

The Convention of quarantine and health officers of the South Atlantic and Gulf States, which convened in Atlanta on the 12th ultimo, performed its work in a manner conspicuous for promptness and harmony. The meeting lasted only one day, with three sessions. All of the South Atlantic and Gulf States, with the single exception of Texas, were represented.

Dr. H. B. Horlbeck, of Charleston, S. C., was chosen president, and prompt organization was effected. A Committee on Resolutions and Plans was appointed, with Dr. Edmond Souchon, of New Orleans, as chairman. The committee reported to the Convention a resolution favoring the appointment by the Federal Government of medical inspectors to be attached to consulates where yellow fever and cholera are epidemic, and urging Congress to appropriate necessary funds to carry the plan into effect, and also a resolution expressing the opinion "that it is the duty of all nations to take measures to eradicate any plague centers from their territory," and requesting our State Department to convey, through proper diplomatic channels to all nations so affected the sense of this resolution. Both resolutions were adopted.

The committee offered an extensive series of rules and regulations governing quarantine, detention camps, disinfection, shipment of merchandise and general transportation by rail and water during the prevalence of yellow fever, and after discussion and modification, the report as amended was adopted. Other infectious and contagious diseases were not provided for, nor did the scope of discussion embrace the important subject of sanitation.

The rules and regulations adopted are based largely on those formulated by the Marine Hospital Service, but they fall short of that clearness and conciseness which distinguish the rules and regulations now in force by that bureau. The verbiage of the Convention rules and regulations and the minutiæ of detail might have been curtailed with advantage. In some places the instructions are vague and apparently contradictory, and often extensive and implied redundancy. The propriety of enforcement is in too many instances left optional with subordinate local officials.

The rules and regulations are to be put in operation and all appliances apparatus and equipment, furnished by the Marine Hospital Service at *Federal expense*, but under the jurisdiction and supervision of local health and quarantine officers, in order that the local officers may be satisfied that they are properly executed and enforced. Just how conflict and confusion of authority is to be avoided, and whether or not the United States Treasury Department will accept dictation from State and municipal health boards is not stated.

If the Caffery quarantine bill now pending before Congress is enacted into law, as it is believed it will be, the work of the Convention in formulating the rules and regulations will, in all probability, count for little.

A form of health certificate to be used by all boards of health in the South Atlantic and Gulf States, and also the States of Tennessee, Arkansas, and Kentucky, and to be accepted as interstate passports by health and sanitary officers and inspectors of those States, was adopted.

The President was authorized to appoint a committee on the permanent organization of the "Southern Health Association," and to call a meeting of the same at such place and time as may seem expedient. Such an organization will do much to stimulate investigation and disseminate useful information pertaining to the domain of sanitary and quarantine matters. C. M. D.

